# Supplementary material for: How to speed up ion transport in nanopores
Source: Nat Commun. 2020 Nov 30;11:6085. doi: 10.1038/s41467-020-19903-6 (PMC7705656; doi:10.1038/s41467-020-19903-6)
Supplement: Supplementary file 1 — Supplementary Information [file 41467_2020_19903_MOESM1_ESM.pdf]

Supplementary Material to: "How to Speed up Ion Transport in Nanopores"

Konrad Breitsprecher, Mathijs Janssen, Pattarachai Srimuk, B. Layla Mehdi, Volker Presser, Christian Holm, and Svyatoslav Kondrat

S1. EQUIVALENT CIRCUIT

To develop the nonlinear charging protocols for nanoporous electrodes, in the main text we often drew inspiration from the equivalent circuit for flat electrodes, which is a conventional RC circuit. This section provides some necessary information to understand the comparisons made there. We do not claim, however, that the simple RC circuit below would be able to describe our supercapacitor's charging in much detail. We refer to ref. [1, 2] for discussions of more accurate circuit models for supercapacitor charging.

We consider a circuit with a capacitor of capacitance C, a resistor of resistance R, and a voltage source with an applied potential $U(t)$, all connected in series (Fig. S1). The Kirchoff law states that

$$R\frac{dQ}{dt} + \frac{Q(t)}{C} = U(t)\,. \tag{S1}$$

The solution to this textbook problem (see ref. [3], page 21) reads

$$Q(t) = Q_0 \exp\left[-\frac{t}{RC}\right] + \frac{1}{R}\int_0^t dt'\, U(t') \exp\left[\frac{t'-t}{RC}\right]. \tag{S2}$$

where $Q_0 \equiv Q(t=0)$ is the charge on the capacitor at time $t=0$.

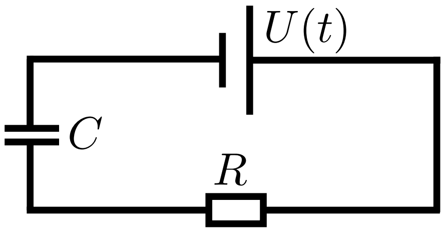

FIG. S1. **Schematic of RC circuit.** The circuit consists of a source providing a time-varying potential $U(t)$, a resistor R and a capacitor C.

A. Step-voltage charging and discharging

For $Q_0 = 0$, charging under influence of a suddenly applied potential $U(t) = U\Theta(t)$ (step-voltage charging) results in

$$Q(t) = Q_{\rm ch}\left[1 - e^{-t/\tau_{RC}}\right], \tag{S3}$$

where $\tau_{RC} = RC$ is the RC time scale and $Q_{\rm ch} = U_{\rm ch}C$ is the accumulated charge at potential $U_{\rm ch}$. We plot these $U(t)$ and $Q(t)$ in Fig. S2 with green lines.

Similarly, discharging the accumulated charge $Q_0 = UC$ by applying a potential $U(t) = U\Theta(-t)$ leads to

$$Q(t) = Q_{\rm ch}\, e^{-t/\tau_{RC}}. \tag{S4}$$

We thus see that step-voltage charging and discharging of an RC circuit happens with the same time constant τ_{RC}. The instantaneous power dissipated over R at the start of the discharge amounts to $P = R(dQ/dt)^2 = U_{\rm ch}^2/R$, and the stored energy is $E = CU_{\rm ch}^2/2$, hence the power to energy ratio gives the time constant, $2E/P = \tau_{RC}$.

B. Linear sweep charging

To complement the above step-voltage expressions, we considered a linear potential sweep,

$$U(t) = \begin{cases} 0 & \text{if } t \leq 0, \\ kt & \text{if } 0 < t < U_{\rm ch}/k, \\ U_{\rm ch} & \text{if } t \geq U_{\rm ch}/k, \end{cases} \tag{S5}$$

shown in Fig. S2 (top plot) for several sweep rates k. Evaluating eq. (S2) for this $U(t)$, we show $Q(t)$ in Fig. S2 (bottom plot) for the same sweep rates. This figure demonstrates that $Q(t)$ is larger for a potential step than for any linear sweep of finite slope.

C. Discharging with Dirac-like voltage-inversion

Finally, we consider a discharging potential sweep of the form

$$U(t) = -2Q_{\rm ch}R\delta(t)\exp\left[-1/\tau_{RC}\right], \tag{S6}$$

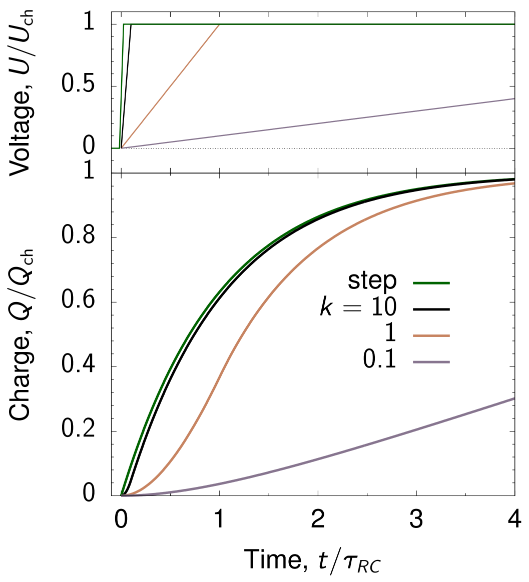

FIG. S2. **Step-voltage *versus* linear sweep charging for RC circuit (Fig. S1).** Charge $Q(t)$ on the capacitor (bottom plot) subject to different time-dependent potentials $U(t)$, eq. (S5), (top plot), for a few values of the sweep rate k. $Q_{\rm ch} = CU_{\rm ch}$ is the accumulated charge at potential $U_{\rm ch}$, and τ_{RC} is the RC time constant.

which corresponds to a sudden drop of the potential at $t = 0$, and setting it to zero for $t > 0$. Inserting eq. (S6) into eq. (S2) gives $Q(t > 0) = 0$. Hence, theoretically, a Dirac delta potential would discharge a capacitor instantaneously. [Note, however, that the associated infinitely large current through a circuit would heat up the resistor, invalidating eq. (S1).]

D. Summary

In the main text, we discuss several aspects of how the behavior of supercapacitors with ultranarrow pores deviates from the above RC-circuit results. The differences can be summarized as follows:

1. The capacitor in the RC circuit charges and discharges with the same time constant τ_{RC} (eqs. (S3) and (S4)), while charging/discharging of a nanoporous supercapacitor is asymmetric (this work and ref. [4]).

2. For the RC circuit, a step-potential provides the fastest charging (Fig. S2), while for a nanoporous supercapacitor, it charges slower than any linear sweep [4].

3. For the RC circuit, the fastest discharging is obtained by a negative delta spike (assuming that a charged state corresponds to a positive potential, and ignoring dissipation). For a nanoporous suercapacitor, we found a minimal charging time for a finite value of $U_{\rm inv}$ and $k_{\rm inv}$ (eq. (10) in the main text); a Dirac delta would correspond to $k_{\rm inv} \to \infty$ and $U_{\rm inv} \to \infty$.

REFERENCES

[1] C. Lian, M. Janssen, H. Liu, and R. van Roij, Blessing and curse: How a supercapacitor's large capacitance causes its slow charging, Physical Review Letters **124**, 076001 (2020).

[2] A. M. AbdelAty, M. E. Fouda, M. T. M. M. Elbarawy, and A. G. Radwan, Optimal charging and discharging of supercapacitors, Journal of The Electrochemical Society **167**, 110521 (2020).

[3] E. Ince, *Ordinary Differential Equations* (Dover, 1956).

[4] K. Breitsprecher, C. Holm, and S. Kondrat, Charge me slowly, I am in a hurry: Optimizing charge-discharge cycles in nanoporous supercapacitors, ACS Nano **12**, 9733 (2018).

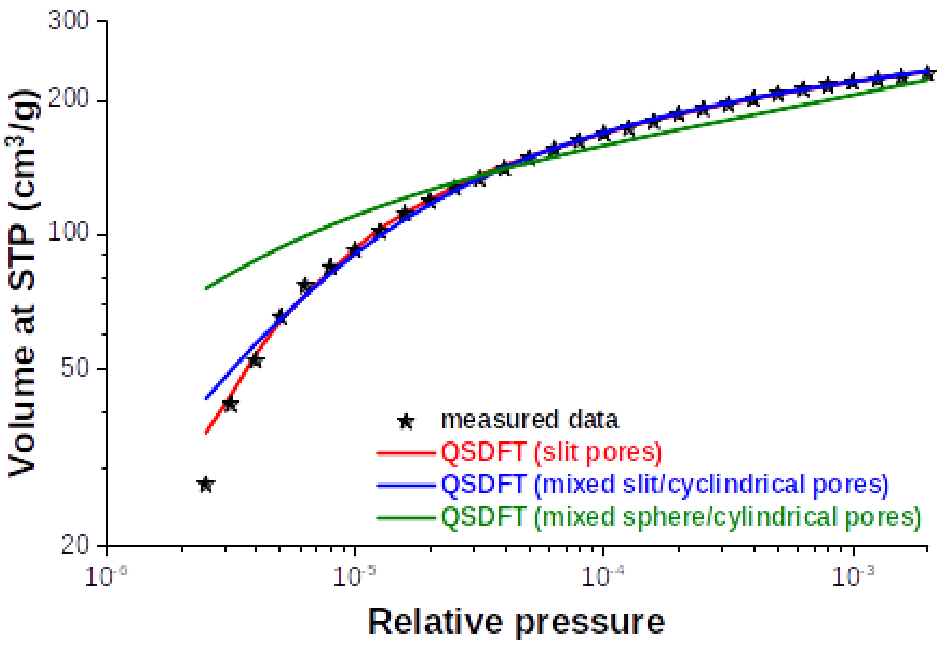

FIG. S3. **Effect of pore shapes.** Adsorbed volume of nitrogen gas (at 77 K) as a function of the relative applied pressure for novolac-derived carbons. Symbols denote the experimental data and the lines show the fit with QSDFT using different assumptions for the pore shape. The best fit is obtained in the case of slit pores.

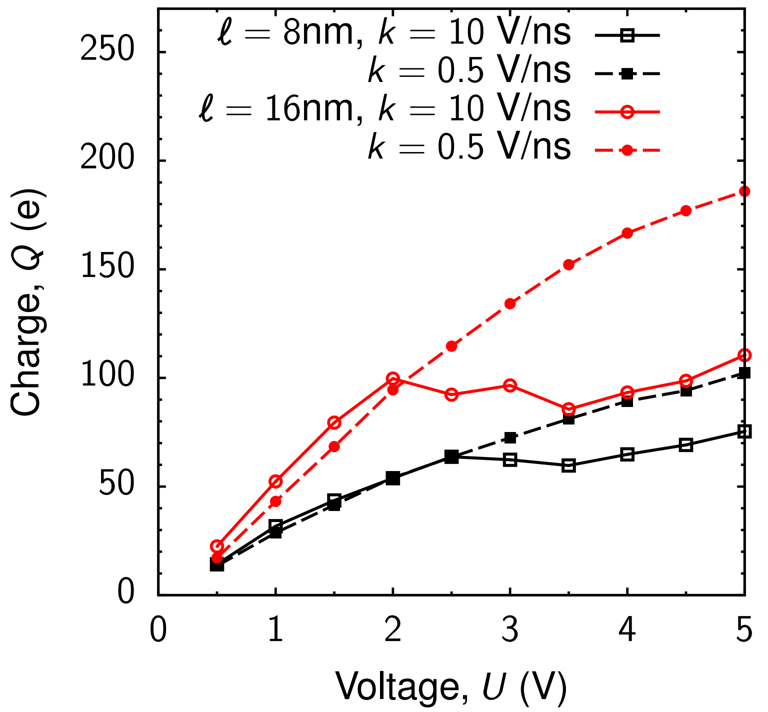

FIG. S4. **Co-ion trapping for different pore lengths.** Charge as a function of applied potential for two pore lengths ℓ using slow and fast charging with sweep rates $k = 0.5\,\mathrm{V\,ns^{-1}}$ and $k = 10\,\mathrm{V\,ns^{-1}}$, respectively. With fast charging the equilibrium charge is not reached due to co-ion trapping. For longer pores co-ion trapping occurs at lower voltages.

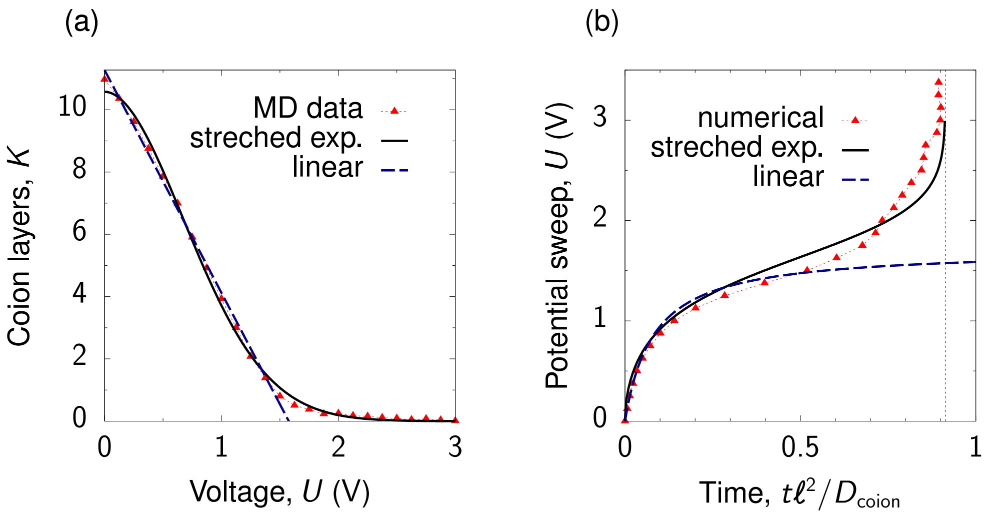

FIG. S5. **Non-linear charging protocol for pore length $\ell = 16\,\mathrm{nm}$.** **a**, Number of coion layers in a pore, K, as a function of applied potential U. Symbols denote the values of K obtained from molecular dynamics (MD) simulations as $K = \rho_c w \ell a = N_c a/h$, where $\rho_c = N_c/(w\ell h)$ and N_c are the density and the number of coions in a pore, respectively, a is the ion diameter, and w, ℓ and h are the width, length and height of the pore (Fig. 1 of the main text); K_0 is the value of K at zero applied potential. The solid line corresponds to the fitting the MD data to the stretched exponential function (eq. (3) of the main text), and the dash line is the fitting to the linear function, see eq. (7) in the main text. Pore width $w = 0.6\,\mathrm{nm}$ and length $\ell = 16\,\mathrm{nm}$. **b**, Nonlinear charging protocols obtained by numerically integrating eq. (2) (main text) with the MD data from panel (a); the stretched exponential approximation (eq. (6) of the main text) and linear approximation for $K(U)$, see eq. (9) in the main text.

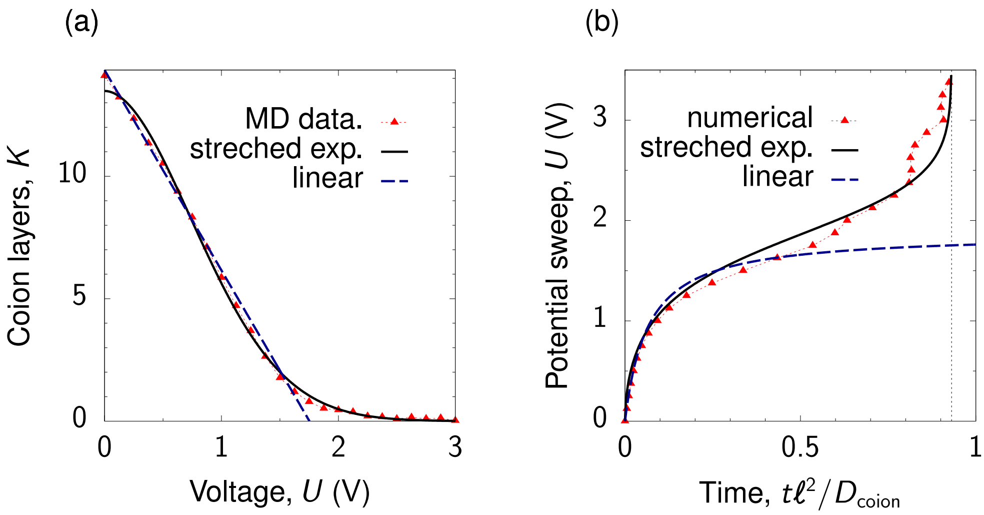

FIG. S6. **Non-linear charging protocol for pore length $\ell = 20\,\mathrm{nm}$. a**, Number of coion layers in a pore, K, as a function of applied potential U. Symbols denote the values of K obtained from molecular dynamics (MD) simulations as $K = \rho_c w \ell a = N_c a/h$, where $\rho_c = N_c/(w\ell h)$ and N_c are the density and the number of coions in a pore, respectively, a is the ion diameter, and w, ℓ and h are the width, length and height of the pore (Fig. 1 of the main text); K_0 is the value of K at zero applied potential. The solid line corresponds to the fitting the MD data to the stretched exponential function (eq. (3) of the main text), and the dash line is the fitting to the linear function, see eq. (7) in the main text. Pore width $w = 0.6\,\mathrm{nm}$ and length $\ell = 20\,\mathrm{nm}$. **b**, Nonlinear charging protocols obtained by numerically integrating eq. (2) (main text) with the MD data from panel (a); the stretched exponential approximation (eq. (6) of the main text) and linear approximation for $K(U)$, see eq. (9) in the main text.

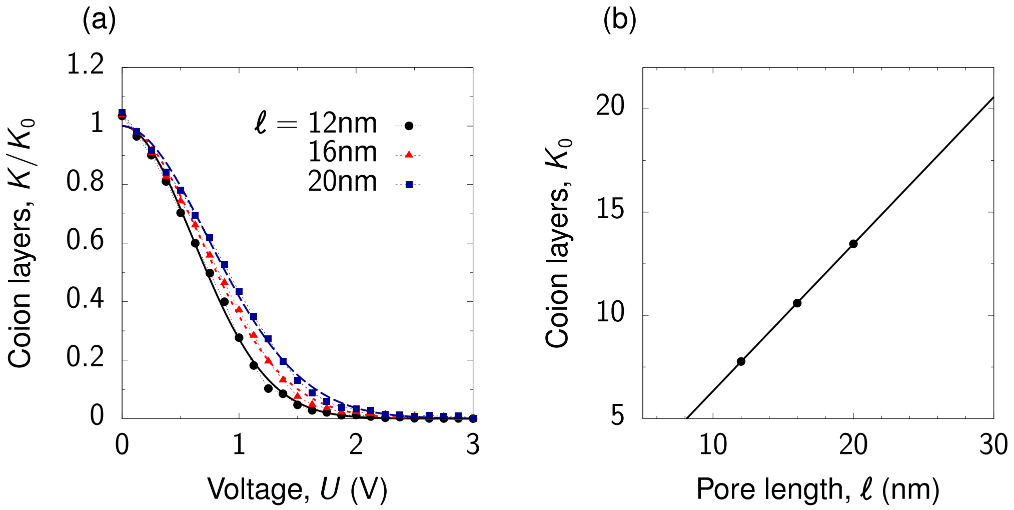

FIG. S7. **Stretched exponential approximation.** **a**, Number of coion layers in a pore, K, as a function of applied potential U for a few pore lengths. Symbols denote the values of K obtained from molecular dynamics (MD) simulations as $K = \rho_c w \ell a = N_c a/h$, where $\rho_c = N_c/(w \ell h)$ and N_c are the density and the number of coions in a pore, respectively, a is the ion diameter, and w, ℓ and h are the width, length and height of the pore (Fig. 1 of the main text); K_0 is the value of K at zero applied potential. The solid lines correspond to the fitting of the MD data to the stretched exponential function (eq. (3) of the main text). Pore width $w = 0.6\,\mathrm{nm}$ and the lengths are indicated on the plot. The fitting parameters are: $K_0 \approx 7.77$, $\alpha \approx 1.96$, $\gamma \approx 1.14\,\mathrm{V}^{-1}$ ($\ell = 12\,\mathrm{nm}$); $K_0 \approx 10.59$, $\alpha \approx 1.91$, $\gamma \approx 1.02\,\mathrm{V}^{-1}$ ($\ell = 16\,\mathrm{nm}$); $K_0 \approx 13.46$, $\alpha \approx 1.91$, $\gamma \approx 0.92$ ($\ell = 20\,\mathrm{nm}$). **b**, Number of coion layers at zero applied potential, K_0, as a function of pore length ℓ. Symbols show the results of the fitting the MD data to the stretched exponential (see panel (a)), and the line shows the result of fitting these values to $K_0(l) = a + b\ell$ ($a \approx -0.78$ and $b \approx 0.71\,\mathrm{nm}^{-1}$).

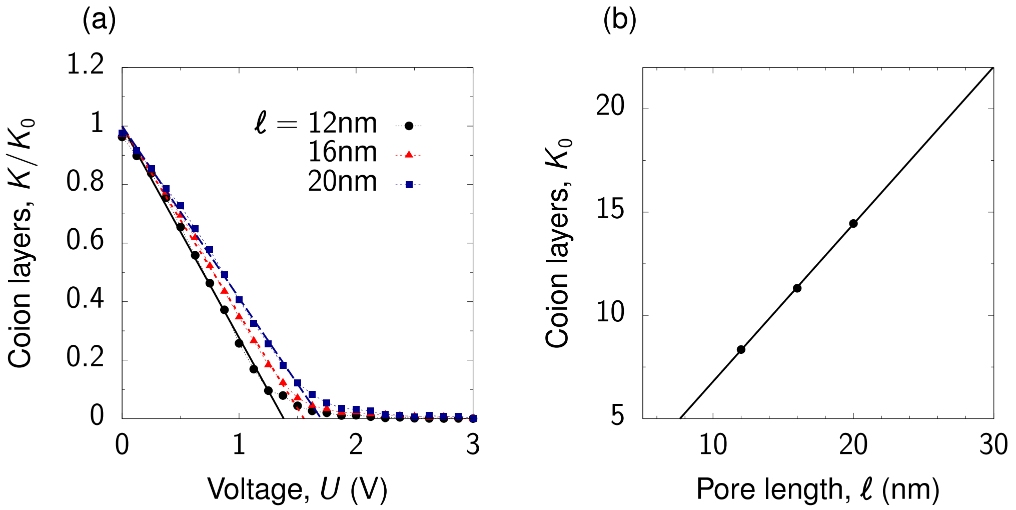

FIG. S8. **Linear approximation.** **a**, Number of coion layers in a pore, K, as a function of applied potential U for a few pore lengths. Symbols denote the values of K obtained from molecular dynamics (MD) simulations as $K = \rho_c w \ell a = N_c a/h$, where $\rho_c = N_c/(w\ell h)$ and N_c are the density and the number of coions in a pore, respectively, a is the ion diameter, and w, ℓ and h are the width, length and height of the pore (Fig. 1 of the main text); K_0 is the value of K at zero applied potential. The solid lines correspond to the fitting of the MD data at low voltages to the linear function (eq. (7) of the main text). Pore width $w = 0.6\,\mathrm{nm}$ and the lengths are indicated on the plot. The fitting parameters are: $K_0 \approx 8.36$, $\gamma \approx 0.72\,\mathrm{V}^{-1}$ ($\ell = 12\,\mathrm{nm}$); $K_0 \approx 11.32$, $\gamma \approx 0.64\,\mathrm{V}^{-1}$ ($\ell = 16\,\mathrm{nm}$); $K_0 \approx 14.45$, $\gamma \approx 0.59$ ($\ell = 20\,\mathrm{nm}$). **b**, Number of coion layers at zero applied potential, K_0, as a function of pore length ℓ. Symbols show the results of the fitting the MD data to the linear function (see panel (a)), and the line shows the result of fitting these values to $K_0(l) = a + b\ell$ ($a \approx -0.83$ and $b \approx 0.76\,\mathrm{nm}^{-1}$).

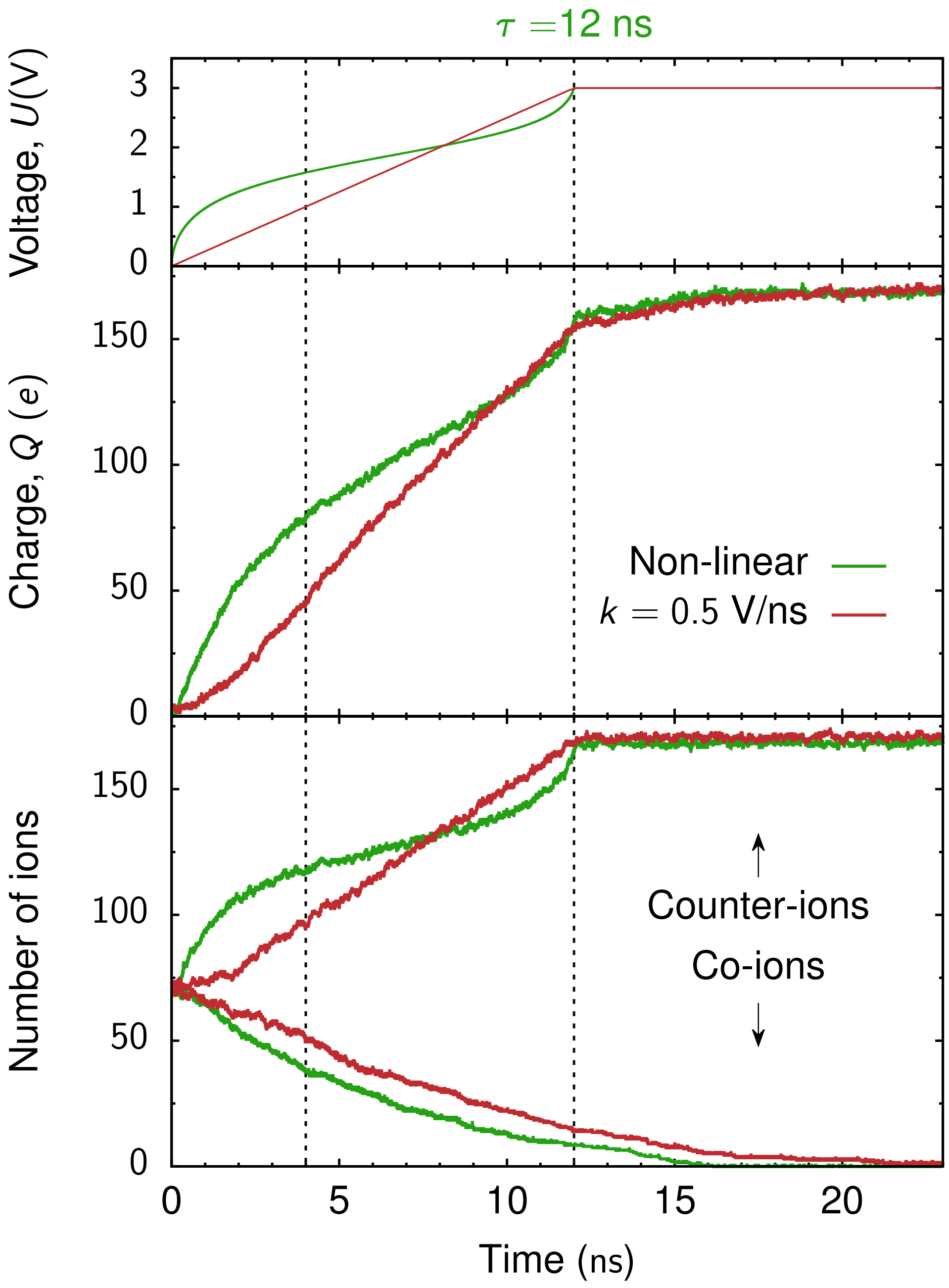

FIG. S9. **Non-linear versus linear sweep.** Both protocols stop at $t = 12\,\mathrm{ns}$, but the nonlinear sweep provides better charging (middle plot). The linear sweep rate $k = 0.5\,\mathrm{V\,ns^{-1}}$ is too fast and leads to stronger co-ion trapping and hence slower co-ion desorption, as compared to the non-linear sweep (bottom plot). For the non-linear sweep, the counter-ion adsorption and co-ion desoprtion times $t_{\mathrm{ads}} \approx t_{\mathrm{des}} \approx 12\,\mathrm{ns}$ (we allow for 4% inaccuracy).

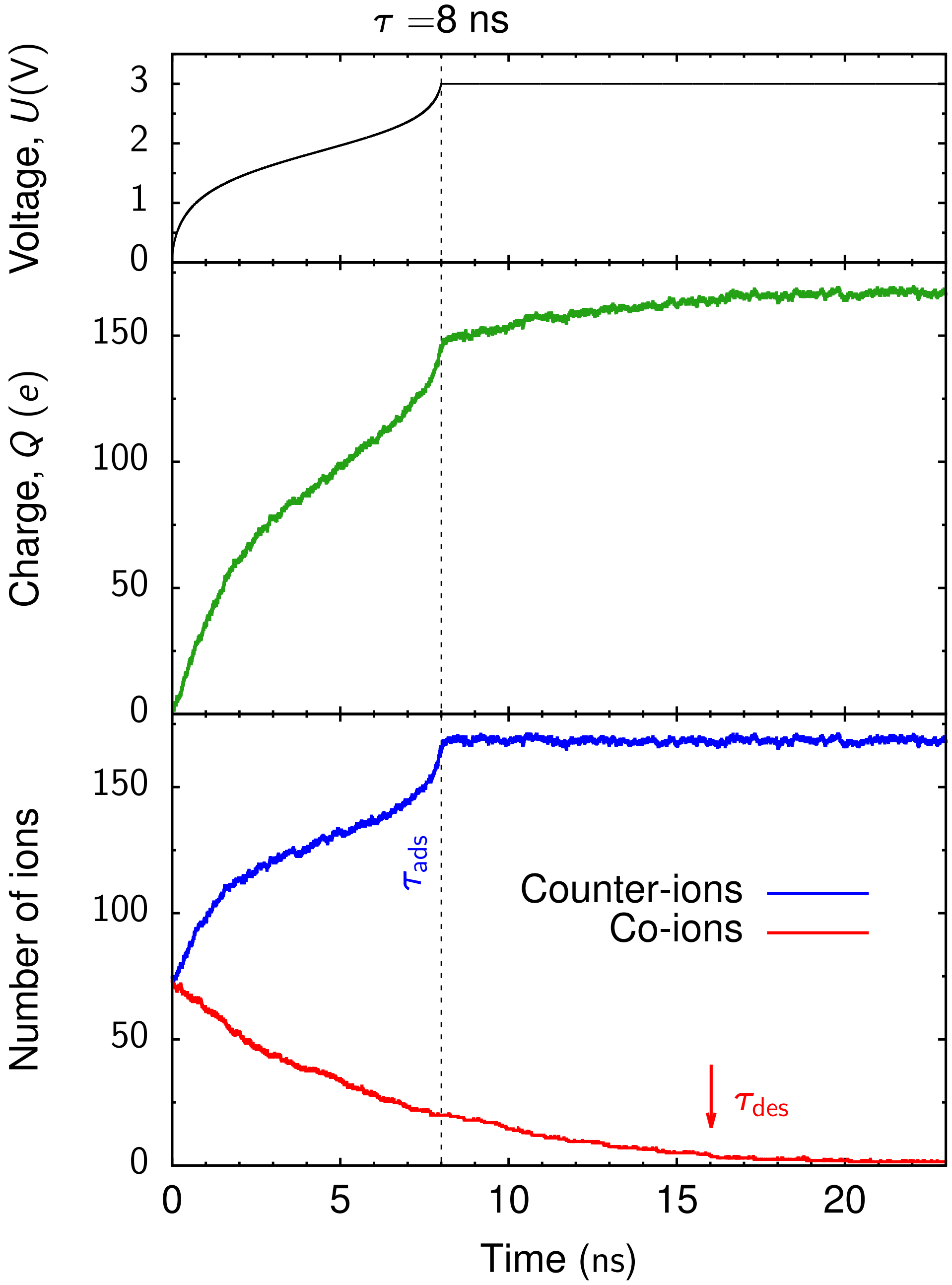

FIG. S10. **Counter-ion adsorption and co-ion desorption times when charging is too fast.** Charging using the non-linear sweep with $\tau = 8\,\text{ns}$ is too fast, which leads to co-ion trapping and large co-ion desorption time t_{des}. The counter-ion adsorption time $t_{\text{ads}} \approx \tau$. Compare with Figs. S9 and S11.

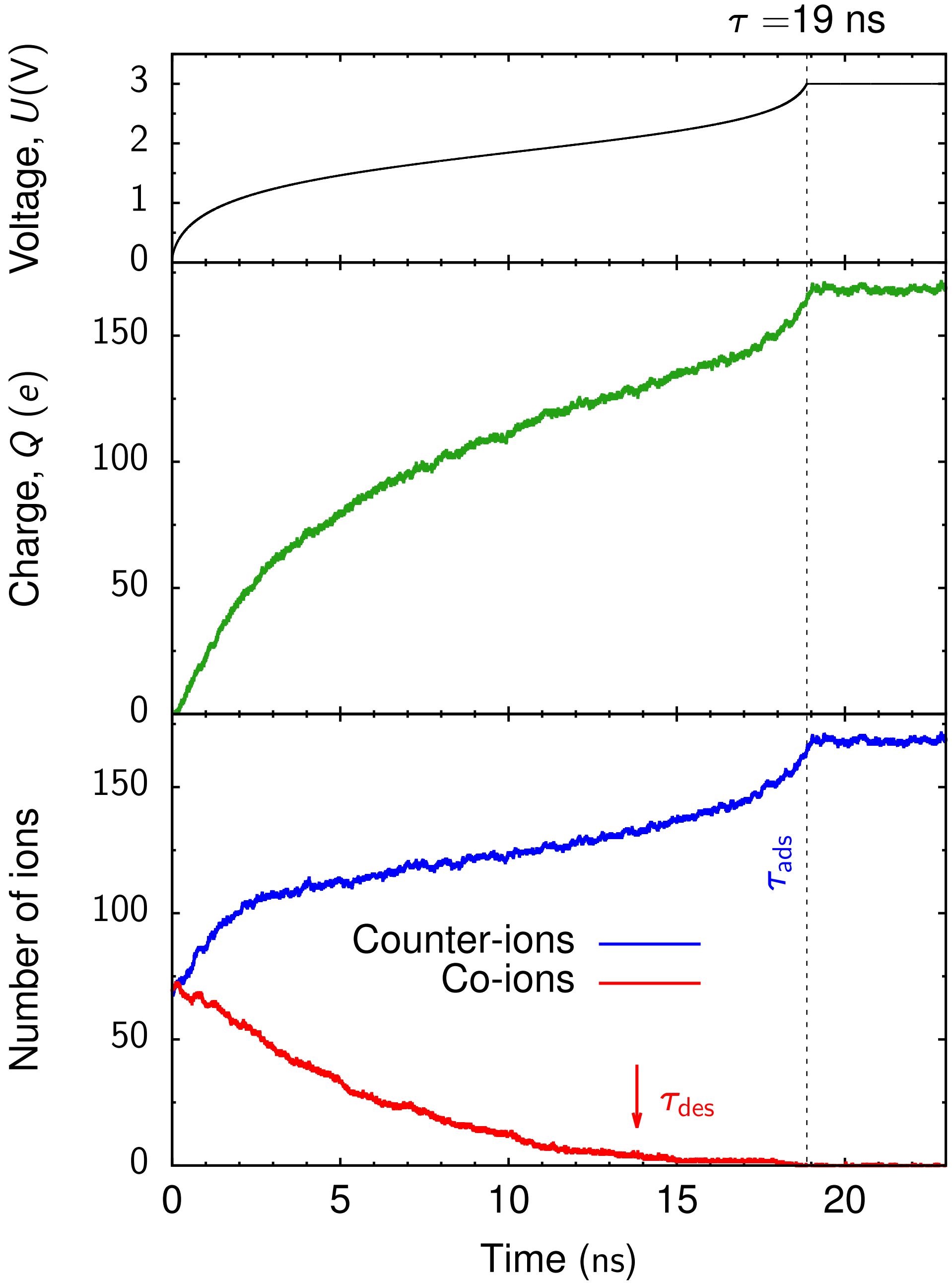

FIG. S11. **Counter-ion adsorption and co-ion desorption times when charging is too slow.** Charging using the non-linear sweep with $\tau = 19\,\mathrm{ns}$ is too slow. There is no co-ion trapping and the co-ion desorption time is short, but the counter-ion adsorption time $t_{\mathrm{ads}} \approx \tau$ is unnessarily large. Compare with Figs. S9 and S10.

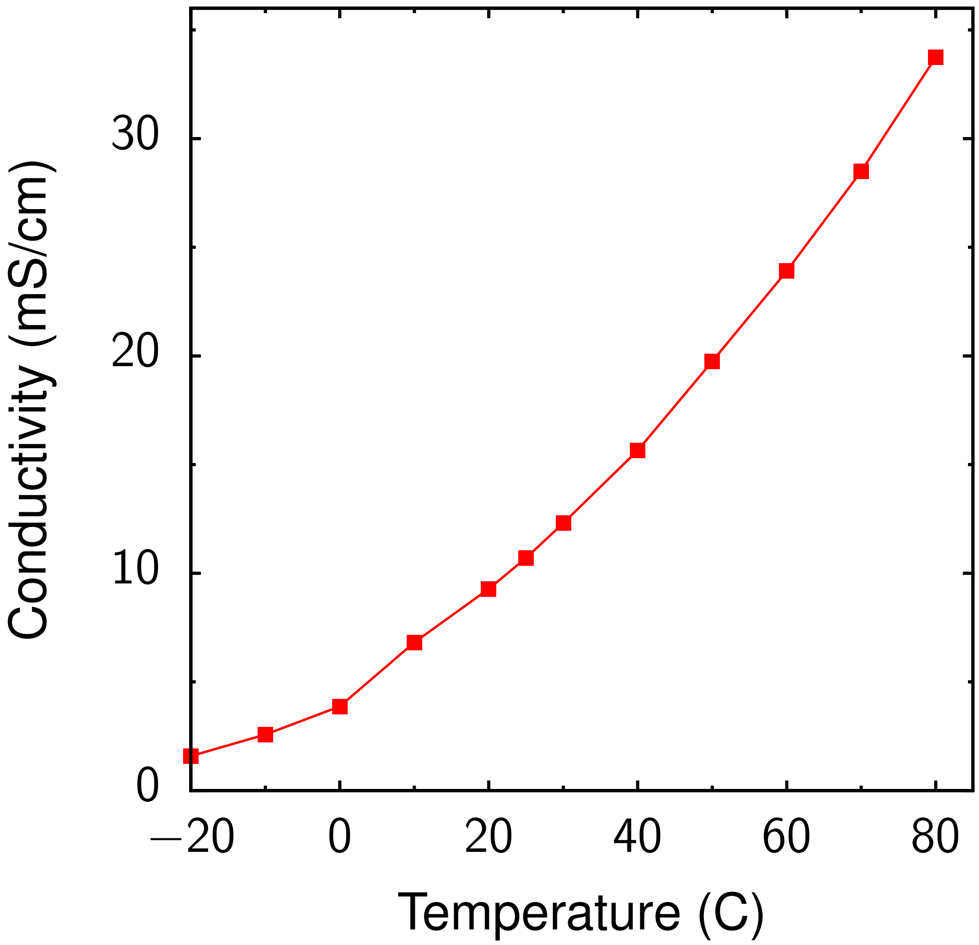

FIG. S12. **Bulk conductivity of EMIM-BF$_4$ ionic liquid.** Conductivity is shown as a function of temperature. At 60 °C the conductivity is about two times higher than at room temperature.

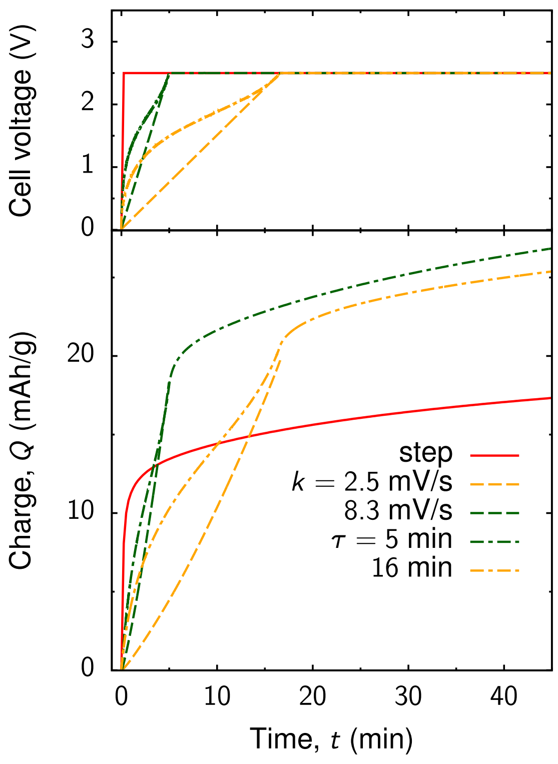

FIG. S13. **Non-linear sweeps versus step-voltage and linear sweeps for novolac-derived carbons.** Cell voltage (top) and accumulated charge (bottom) for step-voltage, linear and non-linear sweep charging. For the non-linear sweeps, cell voltage *vs* time have been obtained by using the stretched exponential approximation (see eq. (3) of the main text), for two values of τ, as indicated on the plot. The linear sweeps have been obtained via a one-cycle voltammetry; the discharging parts of these curves are not shown for clarity. The experiments have been carried out at temperature 60 °C, showing a higher IL conductvity than at room temperature (Fig. S12). Compare with Fig. 4 in the main text. The maximum applied voltage was 2.5 V, instead of 3 V as in Fig. 4 of the main text, because of the IL decomposition occuring at the electrode at higher potential differences.

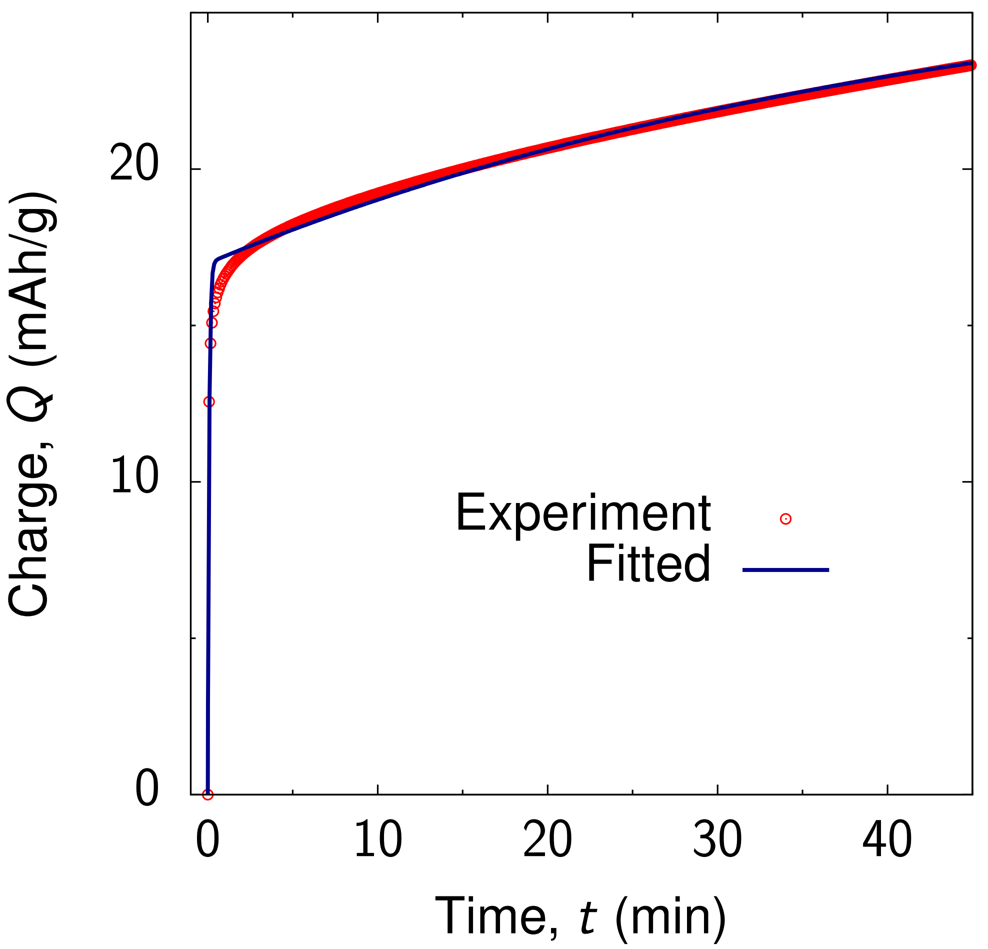

FIG. S14. **Voltage step charging of novolac-derived carbons.** Symbols show the experimental data and the solid line shows the results of fitting the experimental data to function $Q(t) = Q_\infty\,[1 - A_1 \exp(-t/\tau_1) - A_2 \exp(-t/\tau_2)]$ where $A_2 = 1 - A_1$. The fitting parameters are $Q_\infty \approx 27\,\mathrm{mAh/g}$, $A_1 \approx 0.63$, $\tau_1 \approx 0.08\,\mathrm{min}$ and $\tau_2 \approx 45\,\mathrm{min}$.

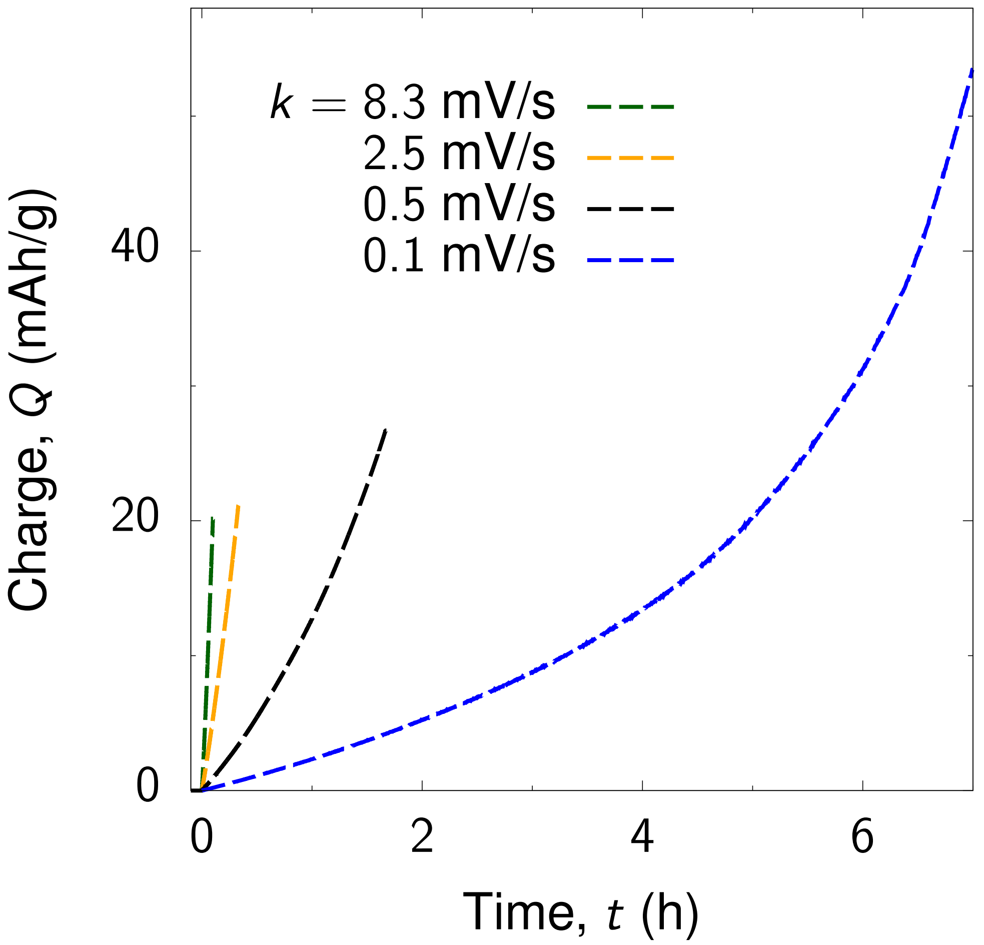

FIG. S15. **Linear sweep charging of novolac-derived carbons.** Charging curves for different sweep rates k. The first cycle of linear sweep voltammetry has been used to obtain the accumulated charge for sweep rates above $k = 0.5\,\mathrm{mV/s}$. Cyclic voltammetry experiments have been used to obtain the results for $k = 0.1\,\mathrm{mV/s}$.

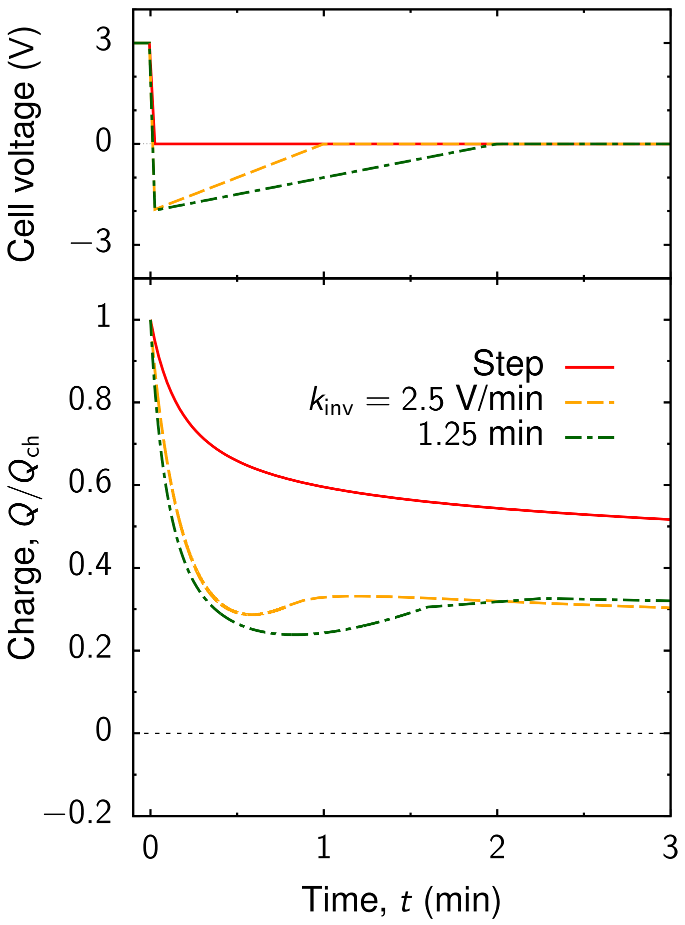

FIG. S16. **Accelerating discharging by voltage inversion for novolac-derived carbons.** Cell voltage as a function of time for step-voltage discharging (red line) and for two voltage-inversion sweeps given by eq. (10) (of the main text) with $U_{inv} = -2\,V$ (top plot). The bottom plot shows the accumulated charge, expressed in terms of the initial charge Q_{ch}, after application of the discharging protocols from the top plot. The supercapacitor was initially charged with a step voltage for about 1 h at cell voltage $U_{ch} = 2.5\,V$. The experiments have been carried out at temperature 60 °C, providing a higher IL conductvity than at room temperature (Fig. S12). Note that discharging is slower as compared to discharging with $U_{inv} = -2.5\,V$ (Fig. 4 of the main text), which is likely because the inversion voltage $-2\,V$ used in this plot was too low.
